# Synthesis and Optoelectronic Properties of Branched Polystyrene-*graft*-Polyfluorene Copolymers

**DOI:** 10.3390/mi17060728

**Published:** 2026-06-16

**Authors:** Chuan Chen, Ruoyu Jiang, Changchun Liu, Pirada Sudprasert, Hong Sun, Guping Tang, Jin Cheng, Kenji Ogino

**Affiliations:** 1 Department of Chemical Engineering and New Material, Changzhou Vocational Institute of Engineering, Changzhou 213164, China; cchen@czie.edu.cn (C.C.); ryjiang@czie.edu.cn (R.J.); ccliu@czie.edu.cn (C.L.); 2 Graduate School of Bio-Applications and Systems Engineering, Tokyo University of Agriculture and Technology, Tokyo 184-8588, Japan; 3 Faculty of Science and Technology, Rajamangala University of Technology Tawan-ok, Chonburi 20110, Thailand; pirada_su@rmutto.ac.th; 4 Zhejiang Fenghong New Material Co., Ltd., Huzhou 313300, China; sunh@zjfhht.com; 5 Department of Chemistry, Zhejiang University, Hangzhou 310028, China; tangguping@zju.edu.cn

**Keywords:** polyfluorene, polystyrene, branched, blue-light stability, electron mobility

## Abstract

Poly(9,9-di-*n*-octylfluorene) (PFO) applications are limited by green emission defects and imbalanced charge transport. To overcome this, novel branched polystyrene-graft-polyfluorene (PSt-*g*-PFO) copolymers with varying grafting densities were synthesized. The highly branched architecture induces intense steric hindrance, acting as a physical shield to isolate PFO emissive cores. This successfully suppresses detrimental interchain π–π stacking, mitigating the ~530 nm green emission. Furthermore, the moderately grafted PSt-*g*-PFO2 promotes locally ordered crystalline packing, achieving a maximum electron mobility of 6.16 × 10^−6^ cm^2^/(V·s), an order of magnitude higher than linear PFO. This structural design effectively decouples deleterious aggregation from charge transport.

## 1. Introduction

Polyfluorene (PF) derivatives, particularly poly(9,9-di-*n*-octylfluorene) (PFO), have emerged as some of the most promising candidates for high-efficiency, pure-blue polymer light-emitting diodes (PLEDs) due to their high photoluminescence quantum yields (PLQY), excellent thermal stability, and superior solution processability [1,2,3,4,5,6,7,8]. However, the practical application of PFO remains hampered by two critical issues. First, the tendency of PFO chains to undergo tight intermolecular π–π stacking in the solid film state often leads to severe interchain aggregation or excimer formation. Combined with keto defects generated during oxidation, this results in an undesirable, parasitic “green-band” emission (~530 nm), which significantly degrades blue-color purity [9,10]. Second, PFO-based materials typically exhibit imbalanced carrier transport, where hole mobility heavily exceeds electron mobility. This imbalance shifts the exciton recombination zone away from the center of the emissive layer, thereby reducing overall device efficiency [11,12].

To address these limitations, various chemical and physical modification strategies have been extensively explored by researchers. Common approaches include the introduction of bulky pendant groups (e.g., dendritic or sterically hindered aryl substituents) to physically suppress interchain interactions, blending PFO with insulating polymer matrices, or synthesizing block copolymers equipped with structurally regulating segments [13,14,15,16,17,18,19].

Among these strategies, the incorporation of non-luminescent, flexible polymer segments, such as polystyrene (PSt), has emerged as a highly effective route due to its excellent insulating properties, thermal stability, and profound ability to modulate solid-state morphology. Inspired by these morphological regulation strategies, in our previous studies, we systematically investigated the effects of incorporating PSt, successfully developing both PFO-*b*-PSt diblock copolymers and PFO-*g*-PSt graft copolymers [20,21]. Our experimental results explicitly demonstrated that the introduction of PSt segments (whether as a terminal block or a grafted branch) effectively promotes the orderly crystalline packing of the PFO backbones, which in turn significantly enhances the electron mobility of the materials. However, while these traditional linear block or random grafting architectures improve charge transport, they often struggle to achieve the complete spatial isolation of the PFO emissive cores while maintaining a low volume fraction of the insulating PSt component, limiting further optimization of spectral stability.

Building upon these prior findings, we propose and synthesize a series of branched polystyrene-graft-polyfluorene (PSt-*g*-PFO) copolymers in this study. These copolymers, featuring precisely regulated grafting numbers (averaging one, two, and three PFO chains per PSt backbone, resembling diblock-like, triblock-like, and three-armed star structures, respectively), were prepared via a mild Steglich esterification by grafting hydroxyl-terminated PFO (PFO-OH) onto a carboxyl-functionalized PSt backbone (PSt-COOH). Unlike traditional linear architectures, this densely grafted branched configuration is designed to generate intense steric hindrance among the crowded side chains. We hypothesize that this spatial repulsion will force the flexible PSt backbone into a compact, sterically congested morphology. Ideally, this unique architecture will act as a robust physical shield to encapsulate the PFO branches, aiming to intrinsically suppress detrimental interchain π–π stacking through molecular conformation rather than microphase separation. To validate this structural strategy, we systematically investigate the impact of this branched architecture on the thermal properties, optical behaviors, crystalline molecular ordering, and electron mobility of the resulting materials.

## 2. Materials and Methods

### 2.1. Materials

N,N,N′,N′,N″-pentamethyldiethylenetriamine (PMDETA), N,N’-dicyclohexylcarbodiimide (DCC), 4-dimethylaminopyridine (DMAP), tetrahydrofuran (THF), dichloromethane (DCM) and other reagents were used as received from Sinopharm Chemical Reagent Co., Ltd. in Shanghai, China. Styrene and 4-vinylbenzoic acid were commercial products from Shanghai Lingfeng Chemical Reagent Co., Ltd. in Shanghai, China. Styrene was distilled under reduced pressure to remove inhibitors and used freshly. CuBr was used after washing with acetic acid and ethanol and drying with nitrogen. The hydroxyl-terminated poly(9,9-di-*n*-octylfluorene) (PFO-OH) was synthesized and purified according to our previously reported procedures [18,19,21].

### 2.2. Characterization and Measurements

Proton nuclear magnetic resonance (^1^H-NMR) spectra were recorded on a JEOL ALPHA300 instrument (JEOL Ltd., Tokyo, Japan) at 300 MHz and 25 °C, using deuterated chloroform as the solvent and tetramethylsilane as the internal standard.

Gel Permeation Chromatography (GPC): The number-average molecular weight (*M*_n_) and polydispersity index (PDI) of the polymers were determined using an Agilent 1260 detector (Agilent Technologies, Inc., Santa Clara, CA, USA), using chloroform as the eluent at 0.5 mL/min. The system was calibrated with narrow-disperse polystyrene standards.

Thermal analysis: Differential scanning calorimetry (DSC) measurements were performed on a Rigaku DSC-8230 (Rigaku Corporation, Tokyo, Japan) under a nitrogen atmosphere. The samples were heated and cooled at a rate of 10 °C/min to determine the glass transition temperature (*T*_g_).

Optical spectroscopy: Ultraviolet-visible (UV-vis) absorption spectra were recorded on a JASCO V-570 spectrophotometer (JASCO Corporation, Tokyo, Japan). Photoluminescence (PL) spectra were measured using a JASCO FP-6500 fluorescence spectrophotometer (JASCO Corporation, Tokyo, Japan) with an excitation at 380 nm.

Grazing-incidence wide-angle X-ray scattering (GIWAXD): The measurements of molecular packing were carried out at the (RIGAKU X-ray Diffractometer SmartLab) (Rigaku Corporation, Tokyo, Japan) (Cu K_α_, λ = 1.5418 Å, 45 kV, and 200 mA) with an X-ray wavelength from 3 ° to 30 ° with a step of 0.02 ° at the scan speed of 1 °/min in the out-of-plane measurements. The incident angle was fixed to 0.14 °.

Electron mobility measurement: The electron mobility (*μ*_e_) was evaluated using the space-charge-limited current (SCLC) method. Electron-only (EO) devices were fabricated on an indium tin oxide (ITO) pattern (10 Ω per square), which were washed with an alkaline cleaner, deionized water and 2-propanol, and dried with nitrogen. EO devices were fabricated with a configuration of ITO/aluminum (Al) (50 nm)/active layer (150 nm)/lithium fluoride (LiF). The current density-voltage (*J*-*V*) characteristics were measured using a Keithley 2400 source meter(Keithley Instruments, Cleveland, OH, USA).

Films Formation: The polymer layer was laminated by spin-coating at 1000 rpm for 60 s from a chlorobenzene solution (40 mg/mL) filtered through a 0.45 μm membrane filter. The films for UV-vis, PL and GIWAXD samples were formed in the same 2.5 cm × 2.5 cm glass plates, and the procedure was almost the same as for EO devices, except for polymer layer formation on ITO substrates. The thickness of the films and metals was measured by a stylus-type surface profiler (BRUKER, Dektak XT-S) (BRUKER Dektak XT-S, Bruker Corporation, Billerica, MA, USA).

### 2.3. Synthesis of PSt-COOH

PSt-COOH was synthesized via atom transfer radical polymerization (ATRP). Fresh distilled styrene (3.12 g, 30 mmol), 4-vinylbenzoic acid (4.41 g, 30 mmol), CuBr (0.86 g, 6.0 mmol), CuBr_2_ (0.066 g, 0.3 mmol), PMDETA (1.26 mL, 3.0 mmol), and 2-bromopropionic acid *tert*-butyl ester (0.94 mL, 6.0 mmol) were placed in a Schlenk flask. The mixture was degassed via four freeze-pump-thaw cycles to remove oxygen. The flask was then heated at 90 °C for 2 h under a nitrogen atmosphere. After the reaction, the mixture was cooled to room temperature, dissolved with sufficient THF, condensed via evaporation, and precipitated dropwise into a large excess of cold methanol. The precipitate was collected by filtration with active alumina, redissolved in THF, recondensed via evaporation, and reprecipitated into methanol twice to remove unreacted monomers. The product was collected by filtration with active alumina and dried under vacuum at 50 °C for 24 h to yield PSt-COOH as a white powder (yield: 51%).

### 2.4. Synthesis of Branched PSt-g-PFO

The branched copolymer was synthesized via a mild Steglich esterification strategy. PSt-COOH (0.13 g, 0.1 mmol, containing 0.5 mmol of -COOH groups), PFO-OH (0.78/1.55/2.33 g, 0.1/0.2/0.3 mmol), DMAP (1.06 g, 8.6 mmol), DCC (1.78 g, 8.6 mmol) and DCM (15 mL) were added into a 50 mL round-bottomed flask. The reaction mixture was stirred at room temperature for 72 h.

After evaporation to remove the DCM solvent, the residue solid was stirred quickly with acetone (60 mL) for 6 h and filtered to remove unreacted non-carboxylated PSt oligomers, along with small-molecule residues (such as DMAP). The crude polymer was subjected to Soxhlet extraction with acetone for 24 h. Finally, the purified polymer was dried under vacuum at room temperature. Pale-yellow solid PSt-*g*-PFO copolymer was collected, yielding 66% (PSt-*g*-PFO1), 69% (PSt-*g*-PFO2) and 71% (PSt-*g*-PFO3), respectively.

## 3. Results and Discussion

### 3.1. Synthesis and Structural Characterization

**Scheme 1:**
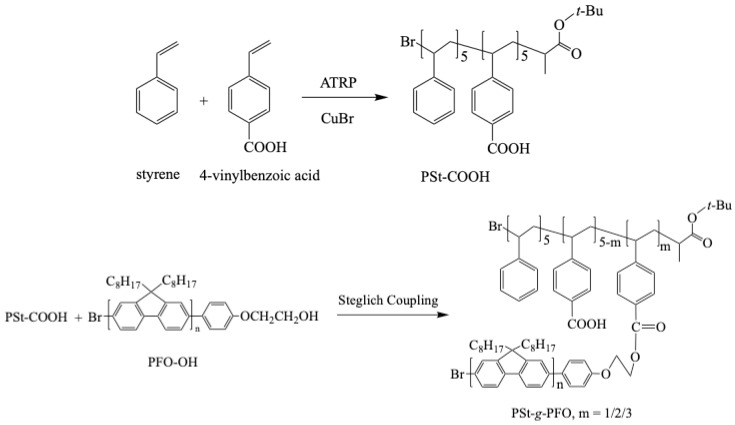
Synthesis route.

First, the carboxyl-functionalized polystyrene backbone (PSt-COOH) was prepared via ATRP. Subsequently, the hydroxyl-terminated PFO (PFO-OH) was grafted onto the backbone using a mild Steglich esterification strategy (Scheme 1).

Notably, conducting ATRP with unprotected carboxyl-containing monomers is generally difficult due to catalyst poisoning via the coordination between -COOH groups and copper complexes. However, in our system, this copolymerization succeeded because we targeted an exceptionally short oligomer (*M*_n_ ~1300 g/mol) and utilized an unusually high catalyst loading (Initiator: CuBr = 1:1). Although the free carboxylic acid groups partially deactivated the catalyst—arresting the polymerization at a moderate yield of 51%—the massive initial excess of active species was sufficient to drive the limited propagation of these short chains. The structural evolution of the copolymers was primarily elucidated by ^1^H-NMR spectroscopy (Figures S1–S5).

The ATRP mechanism was directly verified by ^1^H-NMR (Figure S1). The PSt-COOH spectrum displays characteristic aromatic (6.0–7.2 ppm) and aliphatic main-chain protons (1.2–2.5 ppm). Crucially, end-group analysis based on the terminal methyl signals at 0.8–1.1 ppm and the terminal brominated methine proton (-CH(Ph)-Br) at ~4.5 ppm yielded a *M*_n_ of ~1300 g/mol.

Subsequently, the ^1^H-NMR spectra of the resulting copolymers (Figures S3–S5) provided definitive proof of the successful grafting. The Steglich esterification was confirmed by the emergence of a new peak at ~4.5 ppm assigned to the ester-adjacent methylene protons (-COO-CH_2_-) and the prominent fluorene aromatic protons (7.5–8.0 ppm). Crucially, by comparing the integration ratio of the PSt aromatic protons (6.0–7.2 ppm) to these PFO aromatic protons (7.5–8.0 ppm), the absolute *M*_n_ of the grafted copolymers could be further quantified.

Furthermore, the acetone Soxhlet extraction provided compelling indirect evidence for the PSt-COOH precursor synthesis. Since unreacted short-chain PSt oligomers are highly soluble in acetone, the thorough extraction completely removed any free, ungrafted PSt backbones. The preservation of the characteristic PSt aromatic protons (6.0–7.2 ppm) in the spectra of the purified, acetone-insoluble products definitively proves that the PSt segments are covalently attached to the PFO chains.

Finally, quantitative integration provided definitive proof of the precisely controlled grafting numbers. As detailed in Table 1, the calculated *M*_n_ for the purified copolymers were 9500, 17,000, and 24,200 g/mol. Given that a single PFO-OH chain is ~7800 g/mol, these values impeccably match the theoretical stoichiometric sums of the ~1300 g/mol backbone plus 1, 2, and 3 PFO branches, respectively.

Qualitative evidence of the grafting was also observed via GPC (Figure S6). The elution curves of the PSt-*g*-PFO copolymers shifted to shorter retention times compared to the PSt-COOH precursor, reflecting an increased hydrodynamic volume.

### 3.2. Thermal Properties

**Figure 1:**
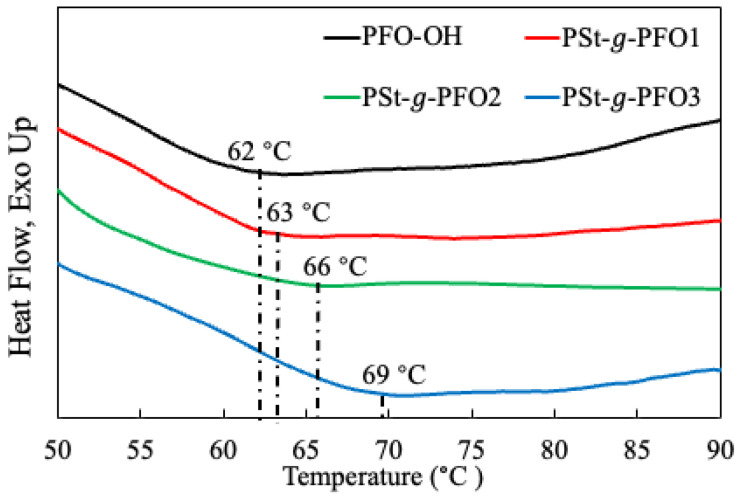
DSC thermograms for PFO-OH and PSt-*g*-PFOs.

The *T*_g_s of PFO-OH and PSt-*g*-PFOs were evaluated by DSC (Figure 1). The *T*_g_ of PFO-OH was measured to be approximately 62 °C. For the branched PSt-*g*-PFO copolymers, the *T*_g_ values exhibited a systematic increase compared to the linear PFO precursor, driven by the intensified steric hindrance between the densely grafted side chains [22]. Specifically, the *T*_g_ values for PSt-*g*-PFO1, PSt-*g*-PFO2, and PSt-*g*-PFO3 were found to be 63 °C, 66 °C, and 69 °C, respectively.

### 3.3. Optical Properties

**Figure 2:**
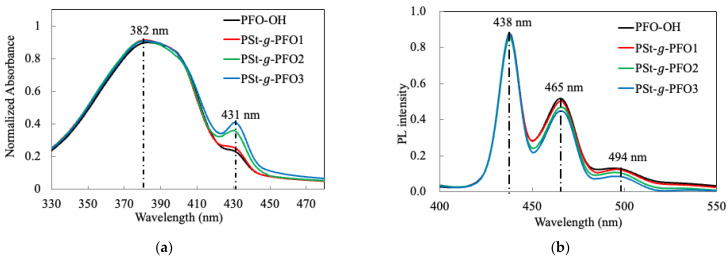
(**a**) UV-vis and (**b**) PL spectra of PFO-OH and PSt-*g*-PFOs.

Solid-state UV-vis and PL measurements were conducted to investigate the optical properties of the polymer films (Figure 2).

Based on the absorption profiles (Figure 2a), the PFO precursor and the grafted copolymers share a dominant π–π transition band centered at 382 nm. A distinct *β*-phase absorption shoulder at 433 nm is also visible across all samples. Interestingly, compared to the pristine PFO, the branched copolymers display an intensified *β*-phase signal that scales with the PFO grafting density. This observation suggests that the spatially congested branched conformation compels the PFO side chains to stretch into a more planar and highly ordered conformation in the solid state [4,23].

The corresponding PL spectra (Figure 2b) further reveal a subtle but meaningful improvement in spectral purity imparted by the graft architecture. All films exhibit typical blue emission signatures, peaking at 438 nm with secondary vibronic bands at 465 nm and a shoulder at 494 nm [24]. However, a closer inspection of the long-wavelength region (~500–550 nm) reveals a gradual reduction in the emission tail. It is well known that linear PFO films are susceptible to a parasitic green emission (~530 nm) driven by strong intermolecular π–π aggregation [25]. While the pristine PFO-OH film exhibits a relatively weak green-band tail in this study, the introduction of the branched PFO segments further suppresses this undesired defect. In alignment with the restricted segmental mobility observed in the thermal analysis, the bulky branched architecture provides a degree of physical shielding. This steric hindrance mildly impedes the close interchain contacts necessary for excimer formation [18,26]. Consequently, by alleviating these aggregation pathways, the structural design helps to preserve and refine the high-purity blue luminescence.

### 3.4. Crystalline Structure Analysis

**Figure 3:**
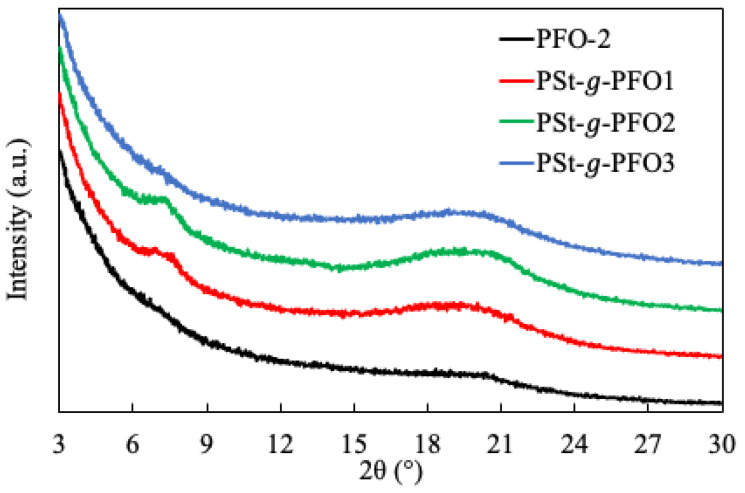
GIWAXD profiles were obtained with out-of-plane geometry for thin films of PFO-OH and PSt-*g*-PFOs fabricated with chlorobenzene.

The solid-state molecular packing of the films was investigated by GIWAXD, as depicted in Figure 3. The pure PFO exhibits a weak, nearly amorphous scattering profile. Copolymers with moderate grafting densities (PSt-*g*-PFO1 and PSt-*g*-PFO2) display distinct diffraction peaks at 2*θ* ≈ 20° (π–π stacking) and 2*θ* ≈ 7.5° (lamellar d-spacing) [27], indicating locally ordered packing. However, for PSt-*g*-PFO3, these diffraction signals weaken noticeably, reverting to a profile similar to the pristine PFO. This attenuation reflects the extreme steric hindrance at the highest grafting density, where the highly congested star-like configuration severely hinders local crystallization. This structural evolution perfectly aligns with the optical findings: the severe spatial crowding inherent to the highly branched architecture fundamentally restricts extensive macroscopic π–π aggregation, thereby assisting in refining the blue emission.

### 3.5. Electron Transport Properties

The electron mobility (*μ_e_*) of the polymer films was evaluated using the space-charge-limited current (SCLC) model. Dark current density-voltage (*J*-*V*) characteristics were fitted to the standard Mott–Gurney equation

(1) $$J=\frac{9}{8}\varepsilon_{r}\varepsilon_{0}\mu_{e}\frac{V^{2}}{L^{3}}$$

where *J* is the current density, *L* is the film thickness, and *V* is the applied voltage (corrected for a 12 Ω series resistance). The relative permittivity (*ε_r_*) was set to 3.5, and the built-in potential was assumed to be zero [28].

**Figure 4:**
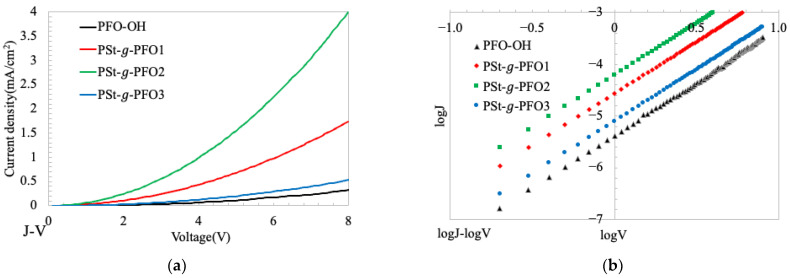
(**a**) Current density-voltage characteristics for electron-transporting devices based on PFO-OH and PSt-*g*-PFOs; (**b**) double logarithmic plots characteristics for electron-transporting devices based on PFO-OH and PSt-*g*-PFOs.

As extracted from *J*-*V* plots (Figure 4) and summarized in Table 2, the pristine PFO-OH exhibited a baseline mobility of 4.13 × 10^−7^ cm^2^/(V·s). For the branched copolymers, *μ_e_* displayed a bell-shaped trend with increasing grafting density. The mobility initially improved, reaching a maximum of 6.16 × 10^−6^ cm^2^/(V·s) for PSt-*g*-PFO2, but subsequently declined to 7.83 × 10^−7^ for PSt-*g*-PFO3.

This transport behavior perfectly mirrors the microstructural transitions. In PSt-*g*-PFO1 and PSt-*g*-PFO2, the moderate grafting architecture induces local ordered packing and robust *β*-phase formation. This optimized morphology creates efficient interchain hopping pathways for electrons. However, at the highest grafting density (PSt-*g*-PFO3), the transport performance deteriorates. The extreme steric hindrance, which yields the highest *T*_g_ (69 °C), substantially disrupts local crystallization. Coupled with the insulating nature of the congested PSt backbone, this structural disruption severely impairs the continuous electron transport network. Thus, an optimal grafting density is vital to strike a balance, simultaneously mitigating parasitic emission while maximizing charge carrier mobility.

## 4. Conclusions

In summary, a series of novel branched PSt-*g*-PFO copolymers with precisely controlled grafting numbers (averaging 1, 2, and 3 PFO chains per PSt backbone) was successfully synthesized via a combination of ATRP and mild Steglich esterification. The structural evolution from a linear precursor to densely grafted branched architectures demonstrated profound effects on the thermal, optical, and electrical properties of the materials.

Thermally, the intensified steric hindrance among the crowded side chains systematically elevated the *T*_g_s for PSt-*g*-PFO2 and PSt-*g*-PFO3. Optically, this bulky branched configuration acted as an effective physical shield in the solid state. By effectively isolating the adjacent fluorene emissive cores, it successfully impeded detrimental intermolecular π–π stacking. This structural barrier suppressed the parasitic green emission defect (~530 nm), thereby preserving exceptionally high-purity blue luminescence across all grafted copolymers.

Furthermore, microstructural and electrical analyses revealed that the electron transport capability follows a bell-shaped trend highly dependent on the grafting density. Copolymers with moderate grafting (PSt-*g*-PFO2) struck an optimal structural balance, promoting robust *β*-phase formation and locally ordered crystalline packing without inducing extreme spatial congestion. Consequently, PSt-*g*-PFO2 achieved the highest electron mobility of 6.16 × 10^−6^ cm^2^/(V·s), which is over an order of magnitude higher than that of the linear PFO precursor. Conversely, excessive grafting (PSt-*g*-PFO3) frustrated local crystallization and severed the continuous electron transport network due to extreme steric hindrance and the insulating nature of the densely packed PSt backbone.

Ultimately, this study demonstrates that dictating the macromolecular architecture through a branched design is a highly effective strategy. By precisely optimizing the grafting density, it is possible to intrinsically decouple deleterious aggregation from charge transport, simultaneously enhancing blue spectral stability and maximizing electron mobility for advanced optoelectronic applications.

## Figures and Tables

**Table 1:** Characteristics of PFO and PSt-*g*-PFOs.

Polymer	*M*_n,PSt-*g*-PFO_ (g/mol) ^a^	PDI ^b^	PFO Branches/PSt	Yields
PFO-OH	7800	1.82	-	82%
PSt-COOH	1300	1.22	-	51%
PSt-*g*-PFO1	9500	1.89	1	66%
PSt-*g*-PFO2	17,000	2.04	2	69%
PSt-*g*-PFO3	24,200	2.17	3	71%

^a^ Determined via ^1^H-NMR. ^b^ Determined via GPC.

**Table 2:** Electron mobility of PFO-OH and PSt-g-PFOs.

Polymer	Electron Mobility (cm^2^/V·s) ^a^
PFO-OH	4.13 × 10^−7^
PSt-*g*-PFO1	2.35 × 10^−6^
PSt-*g*-PFO2	6.16 × 10^−6^
PSt-*g*-PFO3	7.83 × 10^−7^

^a^ Average value determined for four devices.

## Data Availability

The original contributions presented in this study are included in the article/Supplementary Material. Further inquiries can be directed to the corresponding authors.

## Supplementary Materials

The following supporting information can be downloaded at: https://www.mdpi.com/article/10.3390/mi17060728/s1. Figure S1: ^1^H-NMR spectrum of PSt-COOH; Figure S2: ^1^H-NMR spectrum of PFO-OH; Figure S3: ^1^H-NMR spectrum of PSt-*g*-PFO1; Figure S4: ^1^H-NMR spectrum of PSt-*g*-PFO2; Figure S5: ^1^H-NMR spectrum of PSt-*g*-PFO3; Figure S6: GPC plots of PSt-COOH, PFO-OH and PSt-*g*-PFOs.
