# Supplementary material for: Synthesis and Optoelectronic Properties of Branched Polystyrene-graft-Polyfluorene Copolymers
Source: Micromachines (Basel). 2026 Jun 16;17(6):728. doi: 10.3390/mi17060728 (PMC13303755; doi:10.3390/mi17060728)
Supplement: Supplementary file 1 [file micromachines-17-00728-s001.zip › micromachines-4347546-supplementary.pdf]

Supplementary Materials

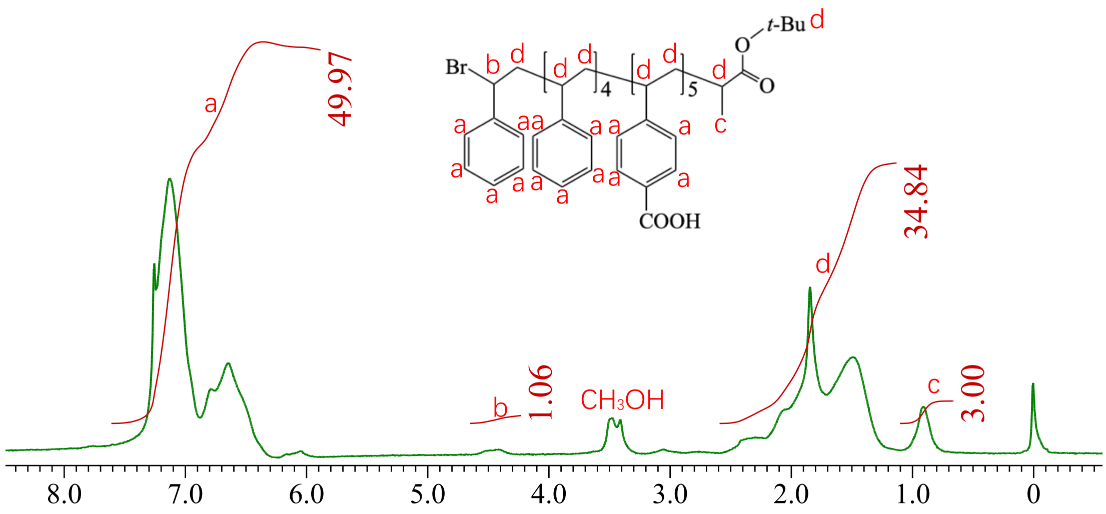

Figure S1: ^{1}H-NMR spectrum of PSt-COOH

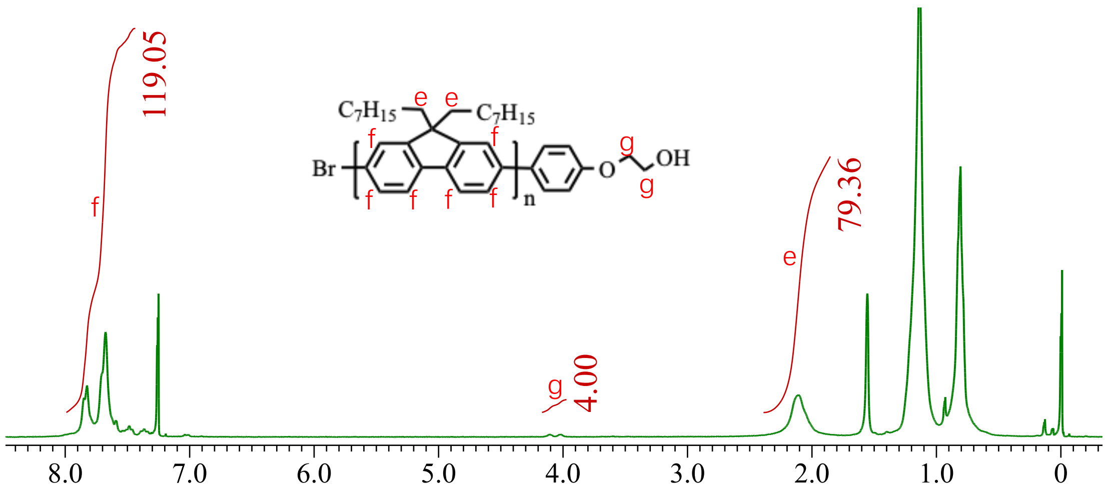

Figure S2: ^{1}H-NMR spectrum of PFO-OH

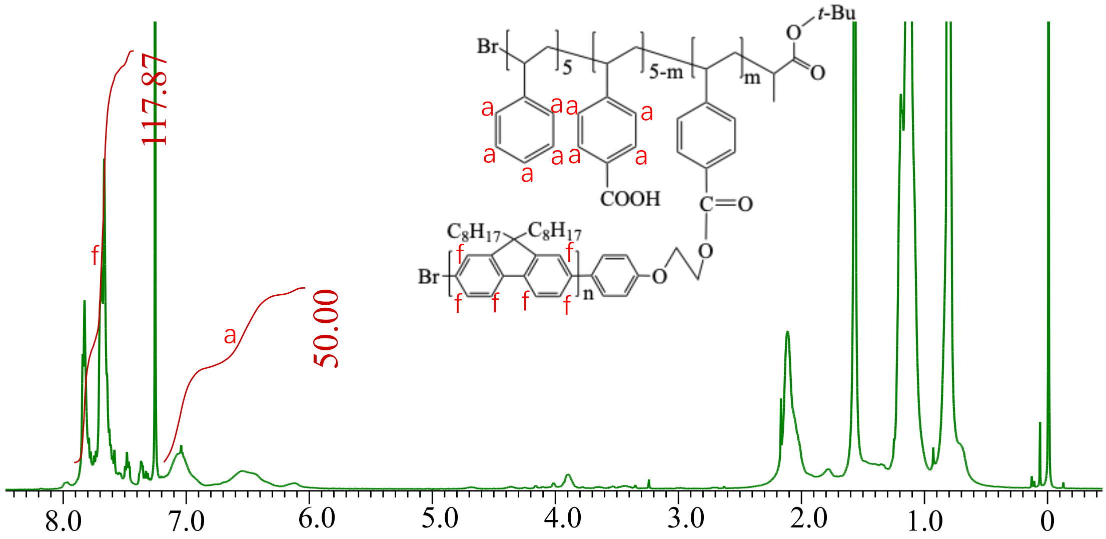

Figure S3: ^{1}H-NMR spectrum of PSt-*g*-PFO1

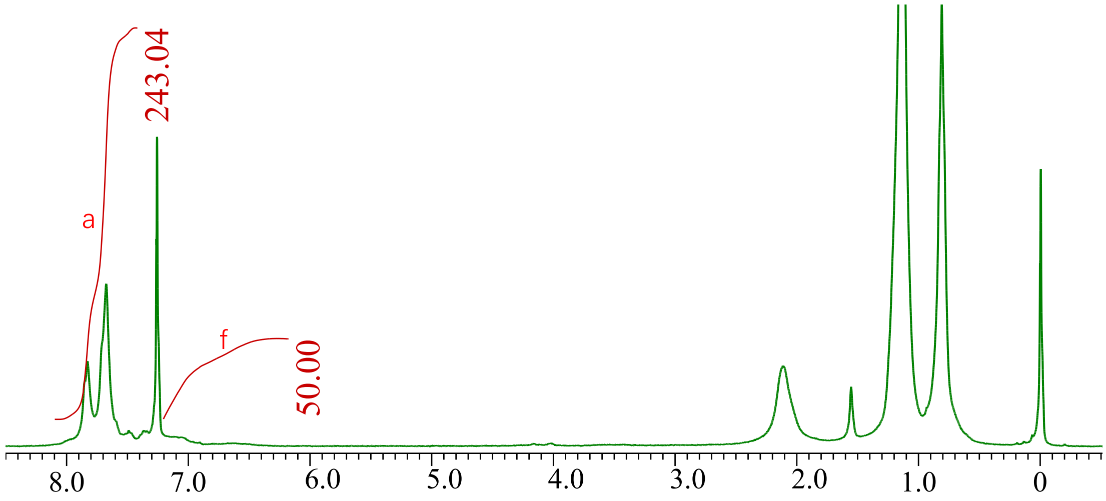

Figure S4: ^{1}H-NMR spectrum of PSt-*g*-PFO2

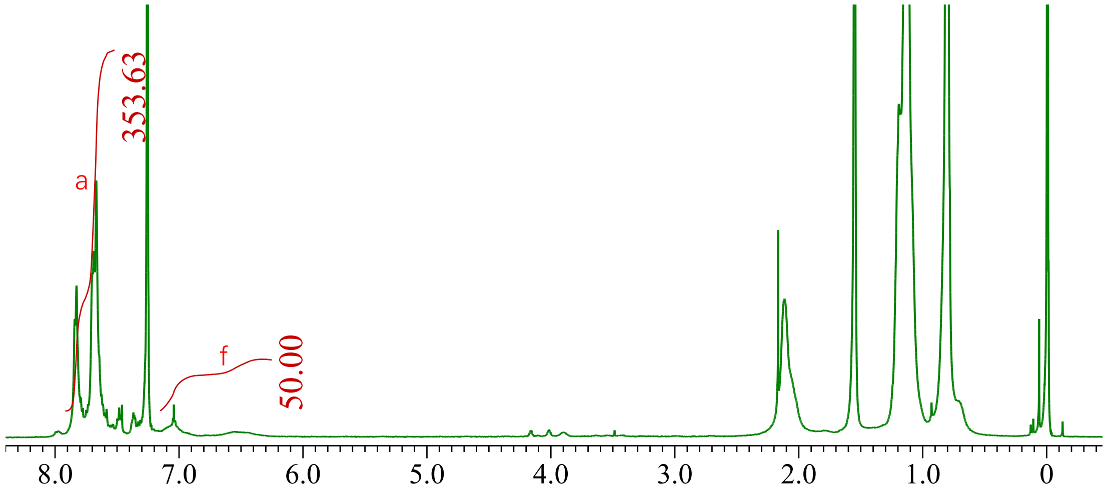

Figure S5: ^{1}H-NMR spectrum of PSt-*g*-PFO3

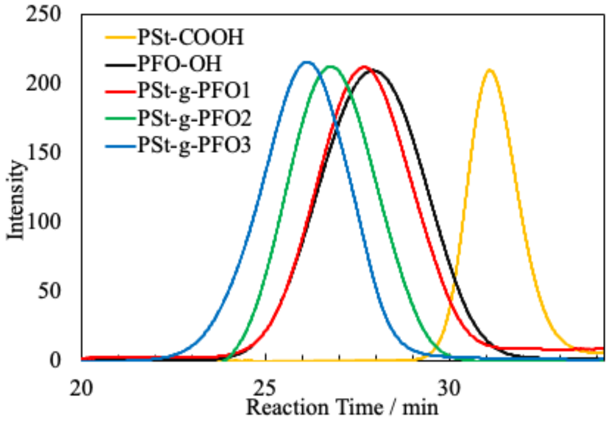

Figure S6: GPC plots of PSt-COOH, PFO-OH and PSt-*g*-PFOs
